# Estimation of Nitrogen Vertical Distribution by Bi-Directional Canopy Reflectance in Winter Wheat

**DOI:** 10.3390/s141120347

**Published:** 2014-10-28

**Authors:** Wenjiang Huang, Qinying Yang, Ruiliang Pu, Shaoyuan Yang

**Affiliations:** 1 Key Laboratory of Digital Earth Science, Institute of Remote Sensing and Digital Earth, Chinese Academy of Sciences, Beijing 100094, China; E-Mails: yellowstar0618@163.com (Q.Y.); yang.shaoyuan@foxmail.com (S.Y.); 2 Key Laboratory of Intelligent Computing & Signal Processing, Ministry of Education, Anhui University, Hefei 230039, China; 3 School of Geosciences, University of South Florida, Tampa, FL 33620, USA; E-Mail: rpu@usf.edu

**Keywords:** winter wheat, nitrogen, vertical distribution, bi-directional reflectance distribution function (BRDF)

## Abstract

Timely measurement of vertical foliage nitrogen distribution is critical for increasing crop yield and reducing environmental impact. In this study, a novel method with partial least square regression (PLSR) and vegetation indices was developed to determine optimal models for extracting vertical foliage nitrogen distribution of winter wheat by using bi-directional reflectance distribution function (BRDF) data. The BRDF data were collected from ground-based hyperspectral reflectance measurements recorded at the Xiaotangshan Precision Agriculture Experimental Base in 2003, 2004 and 2007. The view zenith angles (1) at nadir, 40° and 50°; (2) at nadir, 30° and 40°; and (3) at nadir, 20° and 30° were selected as optical view angles to estimate foliage nitrogen density (FND) at an upper, middle and bottom layer, respectively. For each layer, three optimal PLSR analysis models with FND as a dependent variable and two vegetation indices (nitrogen reflectance index (NRI), normalized pigment chlorophyll index (NPCI) or a combination of NRI and NPCI) at corresponding angles as explanatory variables were established. The experimental results from an independent model verification demonstrated that the PLSR analysis models with the combination of NRI and NPCI as the explanatory variables were the most accurate in estimating FND for each layer. The coefficients of determination (R^2^) of this model between upper layer-, middle layer- and bottom layer-derived and laboratory-measured foliage nitrogen density were 0.7335, 0.7336, 0.6746, respectively.

## 1. Introduction

Nitrogen is a key factor for plant photosynthesis, ecosystem productivity and leaf respiration [1–3]. Determining the optimal amount of nitrogen fertilization to match the demands of crop growth is critical for improving grain yield and reducing environmental impacts [4,5]. Nitrogen stress may affect the light use efficiency and consequently influence long-term changes in vegetation biomass and carbon sequestration [6]. Excessive nitrogen fertilization on farmland can also cause crop lodging, groundwater contamination, atmospheric pollution and other environmental problems [7]. In addition, the transmission of nitrogen in response to nitrogen stress is generally from the bottom-layer to upper-layer in the crop [8]. Nitrogen deficiencies usually exhibit in the bottom layer leaves, while excessive nitrogen will affect the upper layer leaves first. Thus, fertilization should ideally be given according to crop nutrition status as reflected by vertical foliage nitrogen distribution, which is critical for early assessing crop growth status.

Traditional methods of measuring foliage nitrogen, such as the Kjeldahl method are time-consuming and labor intensive [9]. The Soil and Plant Analyzer Development (SPAD) which can obtain chlorophyll meter values is used to measure foliage nitrogen based on the close relationship between foliage chlorophyll and foliage nitrogen concentration [10,11], but the relationship may be nonlinear at high nitrogen levels [12], and consequently the method cannot estimate foliage nitrogen concentration regionally. Remote sensing technology provides an alternative for timely detecting the foliage nitrogen status at large scales.

Many spectral vegetation indices derived from canopy spectra have been used to retrieve foliage and canopy nitrogen status, such as Normalized Difference Vegetation Index (NDVI) and Ratio Vegetation Index (RVI) [13–17]. Bausch *et al.* [18] showed that plant nitrogen status could be effectively assessed by the Nitrogen Reflectance Index (NRI). Daughtry *et al.* [12] proposed a vegetation index named Modified Chlorophyll Absorption Ratio Index (MCARI) and applied it for canopy chlorophyll and nitrogen measurements. To reduce the sensitivity to variation in leaf area index (LAI) and soil background, a combined index, the ratio of Modified Chlorophyll Absorption Ratio Index to the second Modified Triangular Vegetation Index (MCARI/MTVI2), is successfully used to assess the foliage nitrogen [19,20]. Chen *et al.* [20] reported that the Double-peak Canopy Nitrogen Index (DCNI) was a good indicator of nitrogen in winter wheat and corn. Many other indices associated with plant pigments such as Normalized Difference Red Edge index (NDRE) and Red-Edge Chlorophyll Index (RECI) were also used to invert the plant nitrogen. Both Photochemical Reflectance Index (PRI) and Structure Independent Pigment Index (SIPI) were found sensitive to nitrogen treatment [21,22]. Filella *et al.* [23] confirmed that the Normalized Pigment Chlorophyll Index (NPCI) offered a potential way for measuring nitrogen status of wheat. Most of these studies with those indices focus on assessing canopy nitrogen, which normally cannot be a comprehensive approach to assess the crop nitrogen status. In comparison with a single view from vertical canopy, multi-angular observations can acquire more rich plant information by considering more canopy parameters. They have been used to detect foliage disturbances in forest ecosystems and to retrieve chlorophyll vertical distribution in winter wheat [24,25]. Therefore, in this study, we proposed a method for assessing vertical foliage nitrogen distribution in winter wheat by bi-directional reflectance difference function (BRDF) data.

Winter wheat is a major crop in China. A method that can accurately and timely assess a vertical foliage nitrogen distribution at Zadoks 41, Zadoks 65 and Zadoks 73 would be helpful to improve the economic benefits and reduce environment impact in winter wheat. Therefore our objectives for this analysis are to: (1) determine sensitive vegetation indices and viewing angles to estimate foliage nitrogen density for each layer (bottom, middle and top) of winter wheat and (2) select an optical model from three partial least square regression (PLSR) models for assessing foliage nitrogen density at each layer.

## 2. Experimental Section

### 2.1. Experimental Design

Field experiments were conducted in winter at the Xiaotangshan Precision Agriculture Experimental Base in 2003, 2004, and 2007. It is located in Changping district, northeast of Beijing City (40°11′ N, 116°27′ E), China. The soil at the field site is classified as a silt clay loam with a mean annual rainfall of 507.7 mm and a mean annual temperature of 13 °C [26]. The spectral data collected in 2003 were used to develop vegetation indices that were significantly correlated with foliage nitrogen density. The 2007 data were used to establish vertical distribution (for an upper layer, middle layer and bottom layer) nitrogen inversion models, while the data collected from 2004 were used to validate the proposed models. In the experimental base, eight widely cultivated winter wheat varieties with different canopy structures were investigated, including three-erectophile varieties (Jing 411, Laizhou 3279, and I-93), two-planophile varieties (Chaoyou 66, and Jingdong 8), and three-horizontal varieties (Linkang 2, 9428, and Zhouyou 9507).

### 2.2. Data Acquisition

#### 2.2.1 *In Situ* Canopy Reflectance Spectra

An ASD FieldSpec Pro spectrometer (Analytical Spectral Devices, Boulder, CO, USA) with a 25° field-of-view fiber optic adaptor was used to measure the canopy reflectance between 10:00 a.m. and 14:00 p.m. (Beijing local time) under clear sky conditions on 8 May 2003 (Flowering, Zadoks 65). All canopy reflectances were measured at a height of 1.3 m above ground, and a BaSO_4_ calibration panel was used to calibrate radiance and reflectance before and after taking a measurement. From different numbers of fields for each of the eight wheat varieties, a total of 60 spectral measurements were taken. Since the fields of each variety were relatively homogeneous, *in situ* spectra were measured from a plot of about 1 m×1 m per field. Each spectral measurement from a plot was calculated by averaging 20 scans to represent the spectrum for the plot/field for later analysis.

#### 2.2.2. Canopy BRDF Reflectance Spectra

**Figure 1.**
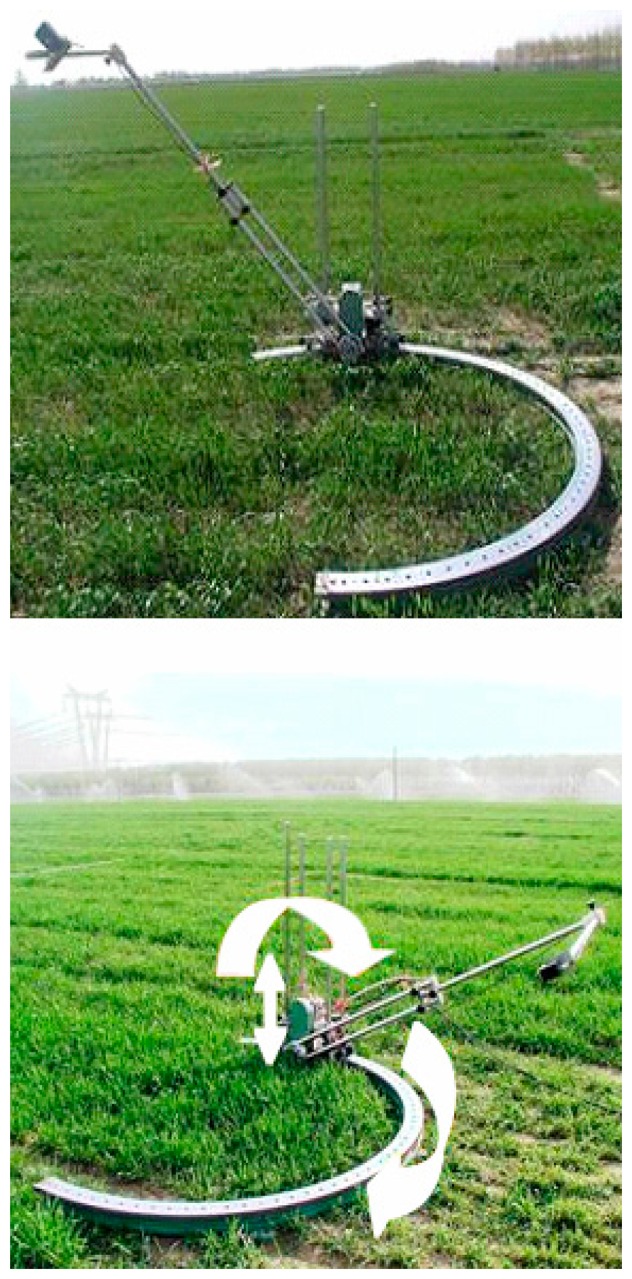
Rotating bracket for observing BRDF canopy reflectance.

The same spectral instrument used for measuring *in situ* canopy reflectance spectra was used to take canopy bi-directional reflectance function (BRDF) reflectance spectra under cloud-free conditions between 10:00 a.m. and 14:00 p.m. (Beijing local time) at a principal plane and a cross-principal plane on 28 April (Booting, Zadoks 41), 9 May (Flowering, Zadoks 65), and 16 May (Milk development, Zadoks 73), in 2004. The similar canopy BRDF reflectance spectra were taken on 28 April, 11 May, and 16 May, in 2007. In accordance with the measuring method introduced by Huang *et al.* [26], a rotating bracket was used to fix the spectral instrument (Figure 1). View zenith angles were from −60° to 60° with 10° intervals. The negative angles represented face-to-the-sun, while the positive angles represented back-to-the-sun. Twenty spectra were taken at each view angle.

#### 2.2.3. Foliar Nitrogen Vertical Distribution and Foliage Nitrogen Density

The vertical foliar nitrogen distribution sampling method used in this study was similar to the vertical foliar chlorophyll distribution sampling method used in Huang *et al.* [25]. The wheat was collected in the plot where the spectral measurement was taken, placed in black plastic bags, and then transported to nearby laboratory. The wheat leaves were separated into three layers (upper-layer, middle-layer and bottom-layer) and the foliage nitrogen concentration of all layers in 2004 and 2007 were determined using the Kjedahl method. Then the 2007 nitrogen density was calculated by using Equation (1) below. Since the leaf dry weight for each layer of 2004 was not measured and only specific leaf weight SLW (g·m^−2^) for each layer was measured, the 2004 nitrogen density was calculated using Equation (2) below:

(1) $$\text{N density}=\frac{\%N\times \text{leafdryweight}}{\text{samplingarea}\times 100}$$

(2) N density=%N×SLW×LAI×100

where, %*N* means nitrogen concentration; LAI means leaf area index. The leaves of each layer were dried at 105° for 10 min and then at 65° for 5 h to obtain the *leafdryweight*.

### 2.3. Data Analysis

A correlation analysis was conducted to examine the sensitivity of vegetation index to foliage nitrogen density. The results were assessed in terms of coefficient of determination (R^2^) between vegetation index and nitrogen density. Correlation analysis was conducted on a dataset of 60 samples collected on 8 May 2003. Nine VIs were first calculated from measured spectra at different view angles according to equations in Table 1. Note that here both MCARI and MTVI2 were combined into a ratio index. These VIs are related to leaf pigment, light use efficiency, and red edge characteristics as aforementioned. R^2^ values between foliage total nitrogen density and the nine commonly used VIs were presented in Table 2. The PLSR was performed to develop multivariate models to measure foliage nitrogen density at three layers. As PLSR algorithm has been described in many studies in detail and the good performance on the agricultural remote sensing has been shown [27,28], so we don't repeat it here. For each layer, three PLSR analysis models were established with three types of vegetation indices as explanatory variables (VIs) and foliage nitrogen density (FND) as a dependent variable. R^2^ and root mean square error (RMSE) were calculated to measure the strength of a relationship between VIs and FND and to assess the accuracy of an estimation of N density.

According to the correlation analysis results of single VIs with FND in Table 2, NDVI showed the best relationship with foliage total nitrogen density (R^2^ = 0.63), followed by NRI (R^2^ = 0.61) and NPCI (R^2^ = 0.60). However, NDVI has been noted to saturate at higher vegetation densities and is insensitive at low densities [13]. We did not consider the three VIs: PPR, DCNI and MCARI/MTVI2 into PLSR modeling analysis due to their R^2^ too low. Therefore, only the remaining six VIs were considered in PLSR modeling analysis. After examining different PLSR modeling results of FND with the six VIs at each vertical layer, three PLSR analysis models were the best for each layer with FND as a dependent variable and three types of VIs (NRI, NPCI or a combination of NRI and NPCI) at corresponding angles as explanatory variables.

## 3. Results and Discussion

### 3.1. Selection of Vegetation Indices (VIs) and View Zenith Angle (VZA)

Based on the results in Table 2 and considering the saturation phenomenon of NDVI, NRI and NPCI might be used to estimate foliage nitrogen density. In order to assess vertical foliage nitrogen distribution, we analyzed the sensitivity of VIs at each view angle to foliage nitrogen density for each layer. We found significant correlations between the FND at the upper layer and NRI at VZAs of 40° and 50°. For the middle layer, there existed significant correlations between FND and NRI at VZAs of 30° and 40°. In addition, the values of NRI at VZAs of 20° and 30° were of linearly significant relation to FND at the bottom layer. Table 3 lists the correlation results of NRI with FND at different view angles at each layer. However, the spectra collected at nadir were the most easily obtained and were used in practice. Therefore, VIs derived (1) at nadir, 40° and 50°; (2) at nadir, 30° and 40°; and (3) at nadir, 20° and 30° view angles were selected to estimate the FND at an upper layer, a middle layer, and a bottom layer, respectively.

### 3.2. PLSR Prediction Models for FND

According to the previous conclusions, the R^2^ values of the three optimal PLSR analysis models were listed in Table 4. It could be inferred from Table 4 that models with a combination of NRI and NPCI as explanatory variables performed the best for each layer compared with other two models.

### 3.3. The Validation of the FND-Prediction Model

**Figure 2.**
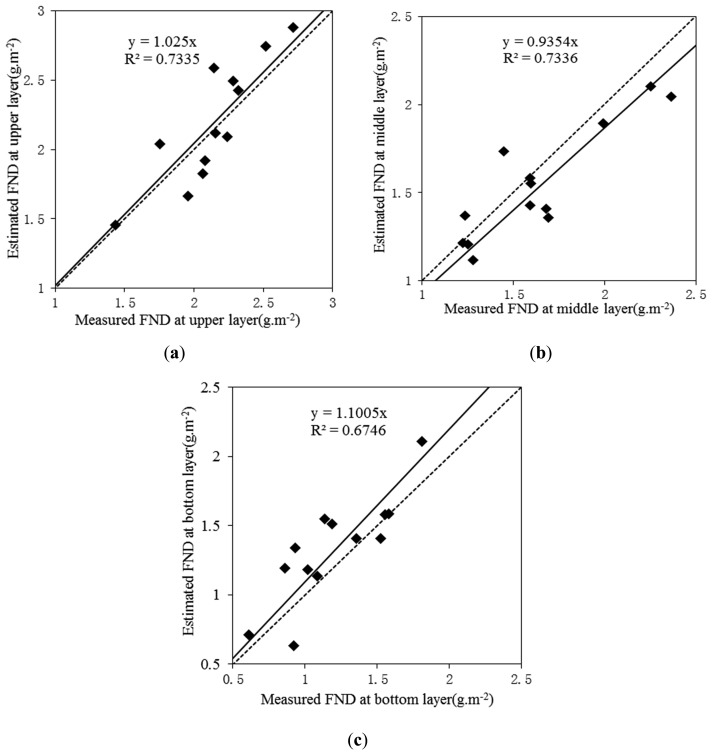
The best validated models (*n* = 13) for each layer: (**a**) upper layer, (**b**) middle layer, and (**c**) bottom layer.

To further test which model can provide the best results for each layer, the data collected in 2004 were applied to validate the three optimal models. By comparison, we found that the PLSR analysis models with the combination of NRI and NPCI as the explanatory variables created the highest R^2^ and lowest RMSE for each layer (Table 5). The best validation results for each layer were shown in Figure 2.

### 3.4. Discussion

In this study, we confirmed that PLSR is an effective statistical approach with multiple view angle observation data for estimating wheat N density. Many previous studies have proven that PLSR is an effective statistical approach. For example, Liu *et al.* [35] demonstrated that PLSR was the most effective approach to predict disease severity compared with other statistical methods. Cho *et al.* [36] reported that PLSR models, based on original, derivative and continuum-removed spectra, produced lower prediction errors compared with NDVI and red-edge position (REP) models. To efficiently utilize spectral information derived from multi-angular remote sensing, in fact, Thomas *et al.* [37] had used their multi-angular high spectral resolution remote sensing data to study crop canopy disturbance at different stages. The method applied in this study that combined PLSR modeling technique with BRDF observation data has further demonstrated the potential of multi-angular remote sensing for extracting information of the vertical foliage nitrogen distribution in winter wheat.

The result from this study has shown that the model with the combination of NRI and NPCI as the explanatory variables is accurate to assess the foliage nitrogen density (FND) at each vertical layer, and it performed best at an upper layer according to the R^2^ and the equations for regression lines. Our results indicated that the model was better if taking more spectral variables (VIs in this time) and multi-angular information into consideration, and that the spectral information from an upper layer (some time also considering a middle layer as in this study) was more easily to record, which is consistent with the point of view of Wang *et al.* [8] mentioned the information from middle layer. The data collected at different view angles contain different layer crop characteristics, and thus multi-angular observations can acquire more rich plant information by viewing relatively full canopy structural characteristics than a single view at a nadir direction. The result from this study is critical for developing a simple method to early assess crop growth status and can also provide a reference for developing multi-angle airborne or space-borne sensors in the future.

However, there were still many shortcomings in this study. Those drawbacks need to be overcome or improved in the future. More experiments should be carried out in different crops by considering different environment factors to further generalize and improve the performance of models. And the effect of crop background (soil, crop residue, *etc.*) reflectance and LAI on spectral response should be considered. Since this study only used the data of observed cross-principal plane, future studies should consider the application of more observation planes such as a combination of cross-principal plane and principal plane for obtaining more comprehensive spectral information from crops. Principal plane contains more information about the plant than other planes, but there are more directional effects than in a cross-principal plane [38]. Other directional factors such as solar zenith angle, solar azimuth angle and view angles also have effects on spectral response [39], and they have not been considered in this paper. However, their influences on crop spectra should be further investigated in future. In addition, our experimental results need to be further calibrated in different areas. Due to limitations of the experimental condition and time, in this study, we did not fully consider the different types of explanatory variables into PLSR analysis models.

## 4. Conclusions

Based on analyses of linear relationships between foliage total nitrogen density and vegetation indices (VIs), Nitrogen Reflectance Index (NRI) and Normalized Pigment Chlorophyll Index (NPCI) were found to be suitable for a retrieval of FND. Further analyzing the linear correlation of NRI at each view angle to FND at each layer, the VIs (NRI and NPCI) (1) at nadir, 40° and 50°; (2) at nadir, 30° and 40°; and (3) at nadir, 20° and 30° were selected to estimate FND at an upper layer, a middle layer and a bottom layer, respectively. Three optimal PLSR analysis models with FND as the dependent variable and three types of vegetation indices (NRI, NPCI or the combination of NRI and NPCI) at corresponding angles as the explanatory variables were established for each vertical layer. The result of an independent model verification demonstrated that the PLSR analysis models with the combination of NRI and NPCI at corresponding angles as the explanatory variables were the most accurate in estimating FND for each layer. The coefficients of determination (R^2^) were 0.73, 0.73 and 0.67, and the root mean square errors (RMSE) were 0.23, 0.19 and 0.24 for the upper layer, middle layer and bottom layer, respectively. This study might provide a basis for using BRDF data and PLSR modeling approach to assess foliage nitrogen vertical distribution over a large area.

## Figures and Tables

**Table 1.** Vegetation indices analyzed in this study.

**Index**	**Name**	**Description**	**Reference**
NDVI	Normalized difference vegetation index	(R_800_ − R_680_)/(R_800_ + R_680_)	Rouse *et al.*, (1974) [29]
NRI	Nitrogen reflectance index	(R_570_ − R_670_)/(R_570_ + R_670_)	Filella *et al.*, (1995) [23]
PPR	Plant pigment ratio	(R_550_ − R_450_)/(R_550_ + R_450_)	Metternicht *et al.*, (2003) [30]
SIPI	Structure insensitive pigment index	(R_800_ − R_445_)/(R_800_ − R_680_)	Peñuelas *et al.*, (1995) [31]
NPCI	Normalized pigment chlorophyll index	(R_680_ − R_430_)/(R_680_ + R_430_)	Peñuelas *et al.*, (1994) [32]
SRPI	Simple ratio pigment index	R_430_/R_680_	Peñuelas *et al.*, (1994) [32]
R810/R560	Ratio vegetation index of 810 nm and 560 nm	R_810_/R_560_	Shibayama and Akiyama (1989) [33]
DCNI	Double-peak canopy nitrogen index	((R720 − R700)/(R700 − R670))/(R720 − R670 + 0.03)	Chen *et al.*, (2010) [20]
MCARI	Modified chlorophyll Absorption ratio index	(R_700_ − R_670_ − 0.2(R_700_ − R_550_))*(R_700_/R_670_)	Daughtry *et al.*, (2000) [12]
MTVI2	Modified triangular vegetation index	1.5(1.2(R_800_ − R_550_) − 2.5(R_670_ − R_550_))/sqrt((2R_800_ + 1)^2^ − (6R_800_ − 5sqrt(R_670_)) − 0.5)	Haboudane *et al.*, (2004) [34]
MCARI/MTVI2	Combined index	MCARI/MTVI2	Eitel *et al.* (2007) [19]

**Table 2.** Coefficients of determination (R^2^) between foliage total nitrogen density and vegetation indices at VZAs of 0°.

**Index**	**R^2^**
NDVI	0.627
NRI	0.610
NPCI	0.604
SRPI	0.603
R810/R560	0.602
SIPI	0.592
PPR	0.0891
DCNI	0.0120
MCARI/ MTVI2	0.0067

**Table 3.** Coefficient of determination (R^2^) between foliage nitrogen density of each layer and NRI at each view angle (*n* = 20).

**Layer**	**Upper Layer**	**Middle Layer**	**Bottom Layer**
**View Angle (°)**			
20	0.515	0.484	0.308
30	0.577	0.566	0.334
40	0.595	0.536	0.260
50	0.581	0.497	0.277
60	0.519	0.411	0.174

**Table 4.** Coefficients of determination (R^2^) of three PLSR analysis models with foliage nitrogen density at each layer as a dependent variable and VIs at corresponding view angles as explanatory variables. The 2007 data were used (*n* = 20).

**Layer**	**Upper Layer**	**Middle Layer**	**Bottom Layer**
**Index**			
NPCI	0.439	0.513	0.327
NRI	0.774	0.608	0.372
NPCI and NRI	0.818	0.642	0.617

**Table 5.** Validation results from the three optimal PLSR analysis models for estimating foliage nitrogen density at each layer (*n* = 13)

**Layer**	**Upper Layer**		**Middle Layer**		**Bottom Layer**	
			
**Index**	**R^2^**	**RMSE**	**R^2^**	**RMSE**	**R^2^**	**RMSE**
NPCI	0.4899	0.358	0.350	0.347	0.328	0.280
NRI	0.5366	0.345	0.627	0.231	0.532	0.265
NPCI and NRI	0.7335	0.225	0.734	0.192	0.675	0.245
